# ControlFace: Feature Disentangling for Controllable Face Swapping

**DOI:** 10.3390/jimaging10010021

**Published:** 2024-01-11

**Authors:** Xuehai Zhang, Wenbo Zhou, Kunlin Liu, Hao Tang, Zhenyu Zhang, Weiming Zhang, Nenghai Yu

**Affiliations:** 1 Department of Cyber Science and Technology, University of Science and Technology of China, Hefei 230026, China; zxh141613683@mail.ustc.edu.cn (X.Z.); lkl6949@mail.ustc.edu.cn (K.L.); zhangwm@ustc.edu.cn (W.Z.); ynh@ustc.edu.cn (N.Y.); 2 Robotics Institute, Carnegie Mellon University, 5000 Forbes Ave, Pittsburgh, PA 15213, USA; haotang2@cmu.edu; 3 Suzhou Campus, Nanjing University, Suzhou 215163, China; zhenyuzhang@nju.edu.cn

**Keywords:** face swapping, feature disentanglement, semantic hierarchy-based feature fusion, controllable identity feature transfer

## Abstract

Face swapping is an intriguing and intricate task in the field of computer vision. Currently, most mainstream face swapping methods employ face recognition models to extract identity features and inject them into the generation process. Nonetheless, such methods often struggle to effectively transfer identity information, which leads to generated results failing to achieve a high identity similarity to the source face. Furthermore, if we can accurately disentangle identity information, we can achieve controllable face swapping, thereby providing more choices to users. In pursuit of this goal, we propose a new face swapping framework (ControlFace) based on the disentanglement of identity information. We disentangle the structure and texture of the source face, encoding and characterizing them in the form of feature embeddings separately. According to the semantic level of each feature representation, we inject them into the corresponding feature mapper and fuse them adequately in the latent space of StyleGAN. Owing to such disentanglement of structure and texture, we are able to controllably transfer parts of the identity features. Extensive experiments and comparisons with state-of-the-art face swapping methods demonstrate the superiority of our face swapping framework in terms of transferring identity information, producing high-quality face images, and controllable face swapping.

## 1. Introduction

Face swapping is a technique that transfers the identity information from a source face to a target face while preserving the identity-independent attributes (e.g., pose, expression, lighting, and background) of the target face. It has been used extensively in entertainment, filmmaking, television, and advertisements, thereby creating amusing effects and elevating visual experiences. Artists leverage this technology for innovative digital art, pushing the boundaries of creativity. In the realm of filmmaking, it facilitates seamless facial replacements between characters, thereby enhancing visual effects. Industries such as e-commerce and beauty employ face swapping for virtual try-ons and makeup experiments, contributing to an enhanced shopping experience. This technique has attracted considerable attention from researchers in the field of computer vision.

The key problem of face swapping technology is identity transfer, i.e., how to precisely and adequately transfer the identity-relevant facial features, comprising both structure and texture, to the target face. Most current methods [1,2,3,4,5] use a pre-trained 2D face recognition network [6] to extract identity features and inject them into a generator to achieve identity transfer. However, due to the difference between face generation and face recognition tasks, the identity information extracted by this network may miss many important facial structure details, like face contours, which can result in swapped faces with structure between the source and target faces. More importantly, previous methods rarely consider the transfer of texture features of the face, such as skin color. We believe that texture, as well as structure, is an important component of face identity information. Such an identity transfer method can lead to huge identity differences between the swapped face and the source face in human visual perception.

Another significant challenge pertains to the development of a more versatile and controllable face swapping methodology. We think that it is essential to provide users with the capability to controllably transfer specific facial attributes according to their preferences. For instance, users should have the option to transfer solely the texture of the source face while preserving the structure of the target face, and vice versa. However, since current face swapping methods can not disentangle the structure and texture of the face, controllable face swapping cannot be achieved.

To effectively address the above problems, we propose a novel face swapping network based on the disentanglement of face features. Inspired by works on face reconstruction [7,8], our method uses two 3D autoencoders to disentangle the facial structure and texture, characterizing them as depth embedding and albedo embedding, respectively. Additionally, we complement them with Arcface embedding extracted by the 2D face recognition network [6] for more information about the internal structure of the face. The combination of these three feature embeddings collectively constitutes the identity representation extracted from the source face. Leveraging this disentanglement approach, we are able to achieve controllable face swapping by extracting a portion of the identity embeddings from the source face and another portion from the target face, thereby transferring the partial identity information we choose.

Simultaneously, we encode the target face image into a latent code $w\in R^{18\times 512}$ using the StyleGAN encoder to preserve its identity-independent information. In order to fuse the structure and texture from the source face and the identity-independent information from the target face together to control and guide the generation of swapped faces, we designed a face feature fusion network. We inject the extracted feature embeddings into the feature mappers according to their semantic levels and fuse them with the identity-independent information to obtain a new latent code $w^{\prime }$ in the W+ space and then generate the swapped faces through the StyleGAN generator.

During the training process, to more effectively disentangle the structure and texture information, we designed three types of training losses: (1) identity-consistent losses used to guide the transfer of identity-related information (structure and texture); (2) attribute-consistent losses used to preserve identity-independent information (expression, pose, and lighting); and (3) ancillary losses used to improve the fidelity of the generated image and facilitate convergence of model training.

Through these approaches, we can accurately transfer the structure and texture of the source face to the target face. In comparison to previous face swapping methods, our method excels in the more adequate transfer of identity information, particularly in terms of facial contours and texture. This results in face swapping results with higher identity similarity to the source face. Moreover, by disentangling structure and texture, our method enables controllable face swapping, allowing users to select the identity information they wish to transfer and expanding its applicability to a wider range of domains.

Overall, our contribution can be summarized in the following three points:

- We propose a new idea for the face swapping task that we can transfer the identity information more adequately and flexibly via identity feature disentanglement, based on which we propose a new high-quality face swapping framework (ControlFace) and achieve controllable face swapping.
- We propose a novel approach for disentangling structure and texture and accordingly propose a semantic hierarchy-based face feature fusion module, where different semantic levels of features are fused to enable the model to efficiently learn these features and generate the swapped faces. Moreover, we designed some loss functions to make the disentanglement more adequate and accurate.
- Extensive experiments demonstrate the effectiveness of our approach to transfer identity information and perform controllable face swapping.

## 2. Related Work

### 2.1. GAN Inversion

The purpose of GAN inversion is to reconstruct the input image as accurately as possible by mapping it to the corresponding latent code. In this way, we can edit the latent code in order to perform the desired image manipulation. There are two key points in this technique: the latent space and the inversion algorithm. StyleGAN [9,10] can generate high-resolution face images with vivid details due to its powerful representation and synthesis capabilities. Its latent space has been proved to have good disentanglement properties [11,12,13,14] and is well suited for feature editing. In addition, some StyleGAN works [15,16] extend the latent space from $w\in R^{1*512}$ to $w\in R^{18*512}$ , obtaining better reconstruction results. Our approach accomplishes the fusion of face features of different semantic levels based on the W+ space of the pre-trained StyleGAN model.

### 2.2. Face Swapping

As a research interest in computer vision, face swapping tasks have a long history. Most of the early face swapping studies [17,18,19] are based on 3D shape estimation for face alignment and feature transfer, which can produce obvious traces of forgery. Most of the GAN-based methods [1,2,3,4,20,21,22,23,24,25] are target-oriented methods, which use an encoder to extract the identity information of the source face and transfer it to the target face. These methods use a discriminator to improve the fidelity of the swapped images. References [1,2,4] obtain the identity embedding from the face recognition model [6], which is injected into the layers of the generator network for fusion. HifiFace [3] adds a landmark obtained from 3D Morphable Model (3DMM) to this identity embedding to complement the identity-related geometric information. References [22,23] represent source and target faces with latent codes via a pre-trained StyleGAN encoder and fuse them in the W+ space according to the semantic level. These methods control the attributes of the target faces through landmarks or segmentation masks. Recently, some face swapping methods based on a diffusion model [5] have been proposed.

However, all of these face swapping methods above only transfer the structure of the source face, neglecting the texture. They generate swapped images with skin colors that are consistent with the target face. E4S [26] is capable of texture transfer, but its feature disentangling approach and its reliance on pre-trained face reenactment models affect its generation quality. Our approach uses a 2D face recognition model [6] and two 3D autoencoders to extract identity information, resulting in more adequate identity transfer and higher-quality, more controllable face swapping.

### 2.3. Feature Disentanglement

Existing face disentangling methods can be classified into parametric and non-parametric methods. Parametric disentangling methods [27,28,29,30,31,32,33] separate face features such as shape, expression, and texture by modeling the face with 3DMM assumptions. Such methods fit the 3DMM parameters via optimization algorithms or use deep neural networks to regress the results on the input images. Non-parametric methods no longer require predefined models and parameters. SFS-Net [34] and Unsup3d [7] perform unsupervised training based on guessing from shading to shape. LAP [8] exploits multi-image consistency in a non-parametric paradigm to disentangle faces into global facial structure and texture features. GAN2Shape [35] and LiftedGAN [36] attempt to disentangle face facial features using 2D GAN. Unlike the above methods, NPF [37] performs 3D face modeling through a neural rendering mechanism and therefore performs better in terms of detail, resolution, and non-face objects.

## 3. Method

**Figure 1:**
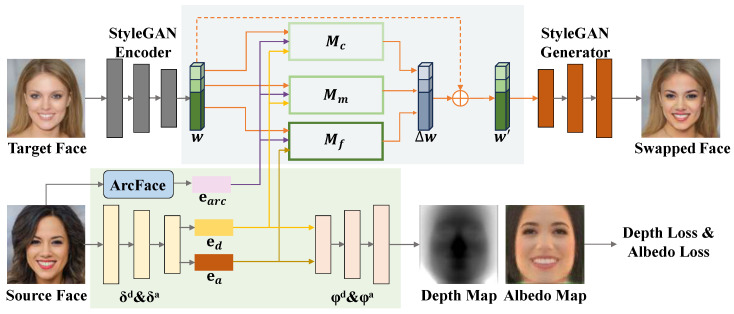
Overview of the proposed ControlFace method. We disentangle and extract the identity information of the source face with two 3D autoencoders and a 2D face recognition model and then represent it as three identity embeddings. Meanwhile, we obtain the latent code of the target face in the W+ space using the StyleGAN encoder. We inject the identity embeddings into the W+ space according to their semantic levels with three feature mappers. Finally, we use the StyleGAN generator to obtain the swapped face.

In this section, we will describe our method ControlFace, which is based on a StyleGAN model. After cropping and aligning the given target and source faces, we first use the StyleGAN encoder to obtain the latent code *w* of the target face in W+ space while extracting the identity embeddings of the source face using two 3D autoencoders and a 2D face recognition model [6]. Then, we inject the disentangled identity embeddings of different semantic levels into W+ space with three feature mappers in the face feature fusion network, obtaining the latent code change Δw . Finally, we input the new latent code $w^{\prime }=w+\Delta w$ into the pre-trained StyleGAN generator to generate the face swapping results. The overall framework is illustrated in Figure 1, and each component will be described in detail below.

### 3.1. Disentangling of Identity Feature

To achieve controllable face swapping, we first need to accurately and adequately disentangle the structure and texture of the source face. Inspired by work on non-parametric 3D face reconstruction [7,8], we use an autoencoder-based approach to disentangle face features. These works use four autoencoders $\phi^{d}$ , $\phi^{a}$ , $\phi^{\omega }$ , $\phi^{l}$ to separate each face image into four parts: the depth map $d\in R_{+}$ , albedo map $a\in R^{3}$ , viewpoint $\omega \in R^{6}$ , and global light direction $l\in S^{2}$ . Such disentanglement is achieved by using the UV relationship of the face features and the basic symmetry principles of structure and texture as follows:

(1) $$\hat{I}=\prod \left(\Lambda \left(a,d,l\right),d,\omega \right),{\hat{I}}^{\prime }=\prod \left(\Lambda \left(a^{\prime },d^{\prime },l\right),d^{\prime },\omega \right),$$

where ∏ and Λ are the illumination and projection steps in the reconstruction process, respectively, and $a^{\prime }$ and $d^{\prime }$ are the flipped versions of *a* and *d*. The method constrains the self-encoders according to the symmetry relationship $I\approx {\hat{I}}^{\prime }$ .

We employ such depth maps and albedo maps obtained from the autoencoders based upon symmetry principles as the representation of structure and texture in our face swapping method. These maps exhibit high identity consistency since facial identity information in the image possesses significantly greater symmetry compared to non-identity information. Specifically, we disentangle the structure and texture of the source face using a depth autoencoder $\phi^{d}=\left(\delta^{d},\varphi^{d}\right)$ as well as an albedo autoencoder $\phi^{a}=\left(\delta^{a},\varphi^{a}\right)$ that are pre-trained. We use the encoders $\delta^{d}$ and $\delta^{a}$ to extract the structure and texture of the source face and represent them as a depth embedding $e_{d}$ and an albedo embedding $e_{a}$ , which are both vectors of dimension 512. Then, we inject these two embeddings into the generative network to guide the face generation. Moreover, we upsample the depth embedding $e_{d}$ and the albedo embedding $e_{a}$ with the decoders $\varphi^{d}$ and $\varphi^{a}$ into the depth map $d_{ref}$ and the albedo map $a_{ref}$ . During the training process, we use these identity feature maps to calculate the identity-consistency loss and guide the face swapping results.

However, since depth maps $d_{ref}$ represent the structure of a face by displaying its distance from the observer in terms of the size of each pixel’s gray value, depth embeddings $e_{d}$ are not sufficient for representing local structure features of the face, especially the eyes, eyebrows, and other detailed parts of the face. Therefore, we still need to complement the source face structure information using ArcFace [6], a 2D face recognition network. We use it to map the source faces into 512-dimensional ArcFace embeddings $e_{arc\;}$ , which will have high cosine similarity if they are extracted from different images of the same identity. Such feature embeddings can effectively complement the information that depth embeddings $e_{d}$ fail to extract in representing facial structure.

Due to the characteristics of the face recognition task, the identity-related information extracted by this face recognition network [6] contains more structure information about the interior of the face, while it is hard for this network to extract texture and face contours effectively. So, we characterize the structure by combining depth embedding $e_{d}$ with ArcFace embedding $e_{arc}$ together while representing the texture through albedo embedding $e_{a}$ .

Based on our disentanglement of structure and texture, our method achieves the capability to perform controllable face swapping tasks. To this end, we devised a multi-mode training strategy that allows the model to learn four modes of identity feature transfer during training, including complete identity transfer, structure-only transfer, texture-only transfer, and self-swapping. When the structure-only transfer is performed, we extract $e_{d}$ and $e_{arc}$ from the source face and $e_{a}$ from the target face. In contrast, when performing the texture-only transfer, we extract $e_{d}$ and $e_{arc}$ from the target face and $e_{a}$ from the source face. When self-swapping, all identity embeddings are extracted from the target face, while they are all extracted from the source face when performing the complete identity transfer. This enables us to achieve four different types of identity feature transfer with the same model, allowing users to choose the identity transfer mode according to their needs. It also enables a small remnant of texture information in $e_{arc}$ to be eliminated during the fusion process, making the feature disentanglement more adequate.

### 3.2. Feature Fusion Based on Semantic Hierarchy

In order to preserve the identity-independent features of the target image and generate high-quality, high-resolution face swapping results, we use the StyleGAN model with powerful representation capabilities. To optimize computational efficiency and enhance training stability, we do not train the StyleGAN model from scratch. For a given target face image, we encode it using the end-to-end StyleGAN inversion method “e4e” [38] into latent codes $w\in R^{18*512}$ in the W+ potential space. Previous face swapping methods [22,23] tend to consider feature fusion only for high-level semantics, but we believe that low-level identity information is equally important for face swapping tasks. For this reason, we designed a multi-level identity injection network for feature fusion.

Many studies [9,39] have shown that the StyleGAN encoder has robust semantic disentangling capability, which means that it is able to disentangle features at different semantic levels of the face image and represent them at different layers in the W+ space, with the more preceding network layers in the model framework corresponding to image information at higher semantic levels. Taking this characteristic of the StyleGAN model as a basis, we separate these layers into three groups (coarse, medium, and fine). Correspondingly, we classify the latent code *w* that represents the image in the W+ space into $w_{c}$ , $w_{m}$ , and $w_{f}$ , which denote high, medium, and low-level semantic features, respectively. In order to fuse identity-related information at different semantic levels in W+ space, we devised three facial feature mappers with the same structure: $M_{c}$ , $M_{m}$ , and $M_{f}$ .

Based on the semantic levels to which different identity feature embeddings belong, we direct their injection into the corresponding mappers. Specifically, the depth embedding $e_{d}$ represents the global structure and contour, with a higher semantic level, and we inject it into $M_{c}$ and $M_{m}$ ; the albedo embedding $e_{a}$ represents the texture, with a lower semantic level, and we inject it into $M_{f}$ . The ArcFace embedding obtained from the 2D face recognition network represents the structure detail information of the internal face, so we inject it into all of the three feature mappers. Therefore, the output of the face feature mapper can be expressed as:

(2) $$\Delta w_{c}=M_{c}\left(w_{c},e_{arc},e_{d}\right),$$

(3) $$\Delta w_{m}=M_{m}\left(w_{m},e_{arc},e_{d}\right),$$

(4) $$\Delta w_{f}=M_{f}\left(w_{f},e_{arc},e_{a}\right).$$

Each mapper consists of 10 units. The first 5 units perform $e_{arc}$ injection, and the last 5 units perform $e_{d}$ or $e_{a}$ injection. In each unit, we further extract the useful parts of the identity embedding through two fully connected networks, especially separating the textures left in the $e_{arc}$ . We fuse them in W+ space according to the following formula:

(5) $$x^{\prime }=\mathrm{LeakyRelu}\left(\left(1+f_{\gamma }\left(e\right)\right)\;\mathrm{LayerNorm}\;\left(x\right)+f_{\beta }\left(e\right)\right),$$

where both $f_{\beta }$ and $f_{\gamma }$ are fully connected networks.

Finally, we use the facial parsing network [40] to predict the face region of the target face and generate the mask, and then we fuse the face region of the swapped face result with the background of the target image using Poisson fusion. In order not to leave visible artifacts at the fusion junction, we performed a soft erosion operation on the generated masks to enable gradient transformations at the blending boundaries of the fused image.

### 3.3. Loss Functions

Our goal is to disentangle and transfer texture and structure from the source face $x_{src}$ while preserving identity-independent attribute information such as expression, pose, and lighting of the target face $x_{tgt}$ . Therefore, we designed several types of loss functions to constrain the generation process of swapped face $x_{awap}$ . In the following, we will introduce the identity-consistency loss, attribute consistency loss, and ancillary loss of our method:

#### 3.3.1. Identity-Consistency Loss

For the $e_{arc}$ extracted from the 2D face recognition network [6], we use cosine similarity to compute the ArcFace loss:

(6) $$L_{arc}=1-\cos\left(e_{arc},R\left(x_{swap}\right)\right),$$

where *R* refers to the 2D face recognition model ArcFace.

In order to transfer the structure and texture of the source image more efficiently, we use the autoencoder $\phi^{d}$ and $\phi^{a}$ to disentangle the source face $x_{src}$ and swapped face $x_{swap}$ into a depth map $d_{ref}$ and albedo map $a_{ref}$ , then compute the depth loss and albedo loss, respectively:

(7) $$L_{depth}={∥\phi^{d}\left(x_{swap\;}\right)-d_{ref\;}∥}_{2},$$

(8) $$L_{albedo}={∥\phi^{a}\left(x_{swap\;}\right)-a_{ref\;}∥}_{2}.$$

Our identity-consistency loss is formulated as:

(9) $$L_{\mathrm{id}\;}=\lambda_{arc}L_{arc}+\lambda_{depth}L_{depth}+\lambda_{albedo}L_{albedo},$$

where $\lambda_{arc}=1$ , $\lambda_{depth}=10$ , $\lambda_{albedo}=10$ .

While performing structure-only transfer or texture-only transfer, we calculate these loss functions according to the specific structure-reference images and texture-reference images of these modes.

#### 3.3.2. Attribute-Consistency Loss

In order to keep the attribute information of the swapped face consistent with the target face, we use an advanced pre-trained 3D face model [32] to parametrically encode the shape, expression, pose, texture, and lighting information of the source face $x_{src}$ and the target face $x_{tgt}$ to obtain the 3DMM coefficients $c_{src}$ and $c_{tgt}$ . Then, we mix the shape coefficients (complete identity transfer and structure-only transfer) and texture coefficients (complete identity transfer and texture-only transfer) of the source face with the other coefficients of the target face to obtain the fused 3DMM coefficients $c_{fuse}$ so that we can obtain the indicative key point coordinates $q_{fuse}$ and the color coefficient $color_{fuse}$ of the swapped face corresponding to it through the mesh renderer and its affine model, respectively. In this way, we can constrain the attribute information of the expression, pose, and lighting by using the landmark loss and color loss:

(10) $$L_{landmark}={∥q_{fuse\;}-q_{swap}∥}_{1},$$

(11) $$L_{color}={∥\;colorfuse\;-\;colorswap∥}_{1}.$$

Moreover, in order to limit the shape change in the swapped face for better foreground-and-background fusion, we designed a segmentation loss:

(12) $$L_{seg\;}={∥M_{swap}-M_{tgt\;}∥}_{1},$$

where $M_{swap}$ and $M_{tgt}$ are the face region masks predicted by the facial parsing network [40] for the swapped face and the target face, respectively.

Our attribute-consistent loss is formulated as:

(13) $$L_{attr\;}=\lambda_{landmark}L_{landmark}+\lambda_{color}L_{color}+\lambda_{seg}L_{seg},$$

where $\lambda_{landmark}=0.1$ , $\lambda_{color}=5$ , $\lambda_{seg}=1$ .

#### 3.3.3. Ancillary Loss

In order to make the model converge faster in the training process, we designed the self-swapping mode, which accounts for 9% of the training steps, in which mode the source face and the target face are the same face image. We calculate the reconstruction loss using the following equation:

(14) $$L_{rec}={∥x_{tgt}-x_{rec}∥}_{1},$$

where $x_{rec}$ denotes the swapped face obtained from the self-swapping mode.

In order to improve the fidelity of the generated images, we used the original discriminator and the adversarial loss function of the StyleGAN2 model, resizing the swapped face images to 256 to input them to the discriminator.

Our ancillary loss is formulated as:

(15) $$L_{anci\;}=\lambda_{rec}L_{rec}+\lambda_{adv}L_{adv},$$

where $\lambda_{rec}=1$ , $\lambda_{adv}=0.05$ . To this end, the total loss of our proposed framework has the following form:

(16) $$L_{total}=L_{id}+L_{attr}+L_{anci}.$$

## 4. Results and Discussion

### 4.1. Experimental Setup

The CelebAMask-HQ dataset [40] contains 30K high-quality 1024 × 1024 face images with great diversity in gender, skin color, and age. We divided it into 27 K and 3 K images, which were used as the training and testing sets, respectively. FaceForensics++ dataset [41] consists of 1000 original videos from the Internet. It serves as the benchmark of many face swapping works and face forgery detection works.

We adopted the same data preprocessing approach as e4e [38], using a pre-trained 68-keypoint detection model to detect the key points of all face images and subsequently cropping and align them and then resize them to a size of 256.

We trained our model on an A6000 GPU. During training, we set the batch size to 16 and used the Adam optimizer [42] with $\beta_{1}$ and $\beta_{2}$ of 0.9 and 0.999 and learning rates of 0.0005 and 0.00005 for the generator and discriminator, respectively. The number of training iterations was 400,000. During the experiment, the inference of our method was about 230 ms/it, around the average level of current face swapping methods.

We compared our approach with previous face swapping methods that have had a large impact, including FaceShifter [1], SimSwap [2], HifiFace [3], InfoSwap [43], and FaceDancer [44]. We also compared with previous StyleGAN-based methods, MegaFS [22] and HiRes [23]. Specifically, we applied all of these methods to the high-resolution CelebAMask-HQ dataset on a test set of 3000 source-target pairs to generate swapped faces. Additionally, we used the two StyleGAN-based methods to conduct an experiment on the low-resolution FaceForensics++ dataset.

### 4.2. Qualitative Evaluation

**Figure 2:**
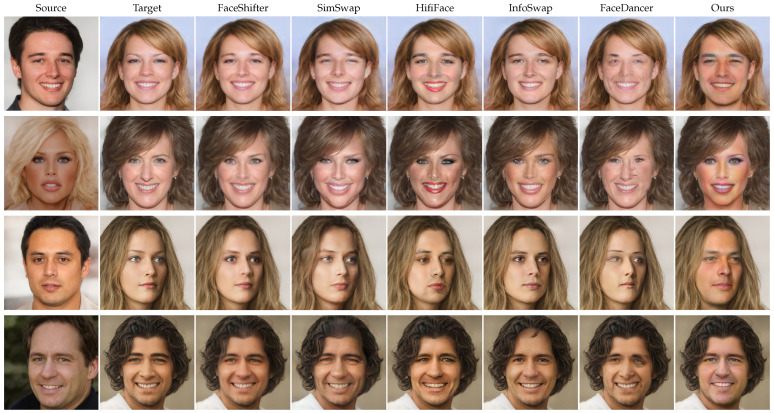
Qualitative comparison of our face swapping results with current state-of-the-art face swapping methods [1,2,3,43,44]. Please pay attention to the texture of the face swapped image.

A qualitative comparison of our method with current state-of-the-art face swapping methods which are not StyleGAN-based is shown in Figure 2. It shows that compared to other methods, our ControlFace method can transfer the identity features from the source face more comprehensively and effectively. It can be seen that FaceShifter [1] is not able to transfer the identity information sufficiently on high-resolution face images, such as the structure of the mouth and nose. FaceDancer [44] also does not perform well in the CelebAMask-HQ dataset, with many artifacts like wrinkles in the face. For SimSwap [2] and HifiFace [3], the detail parts of their results are not processed well enough; especially, the part of the eyes and mouth have more obvious artifacts. Compared to them, swapped faces generated by our model do not have many artifacts. The quality of the swapped face generated by InfoSwap [43] is higher than the methods mentioned before, but compared with our method it is still missing a large amount of important identity-related information, which directly leads to these results having a relatively large identity gap with the source face, especially when there are large differences in contour and skin color between the source and target faces. Among these methods, our method is the only face swapping approach that can transfer texture features and facial contours efficiently. To highlight the advantages of our method, we selected several pairs of faces with significant differences in texture and contour. It can be seen that while dealing with source and target faces with widely varied textures, we are able to transfer the facial color and other texture details of the source face to the target face accurately. For example, for the two faces in the second row, the source face has a darker skin color, while the target face has a lighter skin color. Our method is capable of transferring the skin color from the source face to the target face, ensuring that the swapped face has a skin color as deep as the source face. In contrast, other face swapping methods tend to generate swapped faces with skin colors similar to the target face. Moreover, while dealing with faces in the third row, our ControlFace method is able to transfer the beard from the source face to the target face, while other methods fail to achieve this. In addition, the facial contours of our swapped face results are more consistent with the source face, especially when the source and target faces have large differences in face shapes. This leads to our swapped face results having higher identity similarities to the source face than others.

**Figure 3:**
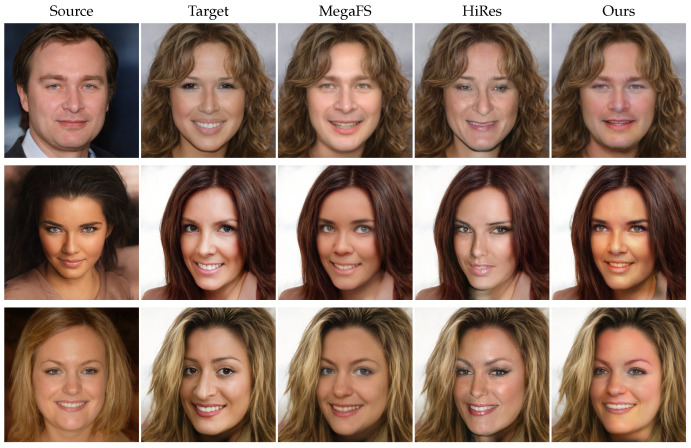
Qualitative comparison of our face swapping results with StyleGAN-based face swapping methods [22,23]. Our method can transfer more identity features than others (facial contours and skin color).

To further compare the performance of our model, we conducted comparative experiments with popular StyleGAN-based face swapping methods. The qualitative results are shown in Figure 3. It is evident that HiRes [23] is unable to transfer the contours and textures of the source face, and the generated images exhibit some artifacts around the mouth area. In contrast, MegaFS [22] can transfer certain textures but fails to disentangle them from the lighting, resulting in blurry and less realistic generated images. Moreover, it struggles to transfer contours effectively. Our ControlFace method, compared to the other two StyleGAN-based methods, excels in transferring both structure and texture. For example, in the first row, the contour of the source face is wider than that of the target face. The swapped faces generated by the other two methods maintain contours almost identical to the target face, while our method produces swapped faces with wider contours, resembling the source face more closely. In the results of the second row, there are noticeable patches of lights on the target face. MegaFS fails to capture this during texture transfer, resulting in facial skin tones with minimal variation in brightness. In contrast, our generated faces are brighter in the lighted areas, demonstrating our model’s ability to transfer texture while preserving the lighting characteristics of the target face.

### 4.3. Quantitative Evaluation

We also conducted a quantitative comparison with the leading methods to compare the ability to transfer source face identity information and preserve target face attributes. We use the face recognition model [6] to extract the $e_{arc}$ of each swapped face and its corresponding source face and calculate the cosine similarity between them to calculate the accuracy rate of identity transfer. Furthermore, we used the depth autoencoder $\phi^{d}$ and the albedo autoencoder $\phi^{a}$ to separate the depth map and albedo map of the swapped face and the source face and compute their $l_{2}$ distances as an indicator of depth error and albedo error to estimate the transfer of structure and texture, respectively. Moreover, in order to quantitatively calculate the preservation of each face feature, we used a 3D face model [32] to extract the shape, texture, expression, pose, and lighting coefficients of each swapped face and the corresponding source face and target face. We computed the $l_{2}$ distances of shape coefficients and texture coefficients between the swapped face and the source face and the other coefficients between the swapped face and the target face.

As can be seen from Table 1, it is evident that our method indeed surpasses current mainstream approaches in the comprehensiveness and accuracy of identity transfer. Our method has the highest ArcFace similarity, which indicates that the swapped faces generated by our method have a high identity transfer rate based on face recognition models. For depth error, the generated results of our method are slightly higher than other methods, which is due to the fact that we are able to transfer the source face contours better. The albedo error of our results is higher than other methods, which shows that our model has better results in transferring the source face texture information, while most of the other methods do not pay much attention to texture transfer.

As can be seen from Table 2, our model achieves near-top results for each face feature. For the shape and texture transfer, our ControlFace method is also more accurate than the others. For attribution preservation, our method achieves third place for expression control and second place for pose control; such a result is mainly based on the landmark loss we propose. The disentanglement of texture and light is currently a significant challenge in texture transfer, due to the fact that they both act together on every pixel value. Nevertheless, our model still manages to maintain low error in light, while ControlFace is the only method that transfers texture in the experiment. This enables our swapped faces to have a high fidelity.

### 4.4. Comparison on FaceForensics++ Dataset

To test the robustness of our model on low-resolution images, we conducted an experiment on the FaceForensics++ dataset [41]. We uniformly selected 10 frames from each video and performed face swapping according to the identities specified by the dataset. To control variables, we compared our results with MegaFS and HiRes, both based on StyleGAN and trained on high-resolution face datasets. We calculated the identity retrieval with the 2D face recognition model and depth and albedo error with 3D autoencoders. The quantitative results of the experiments are shown in Table 3:

We can see that all the StyleGAN-based methods perform worse on the low-resolution dataset. This is because these face swapping methods are all based on StyleGAN2 trained on high-resolution images as the baseline. Nonetheless, our approach still outperforms the other two StyleGAN-based face swapping methods and is able to transfer some of the texture. In the future, we will further optimize the model to enhance its robustness, improve its ability to transfer identity information in low-resolution images and generate high-quality face images.

### 4.5. Ablation Study

We verified the effectiveness of the identity embedding selection and feature injection of our proposed method using an ablation study. We also tested how the size of the training dataset influences the accuracy of our model. In each experiment, we only changed one of the components in our framework to keep the remaining variables constant. The identity transfer capabilities of each model were tested, and the quantitative results are shown in Table 4.

**Choice of identity embeddings:** Our identity extraction network extracts a total of three identity embeddings $e_{arc}$ , $e_{d}$ , and $e_{a}$ . To demonstrate the necessity of individual identity embeddings, we reduced 1–2 identity embeddings at a time and retrained the model. We reduced $e_{arc}$ , $e_{d}$ , $e_{a}$ , and both $e_{d}$ and $e_{a}$ . When we did not inject $e_{d}$ or $e_{a}$ into the feature fusion network, we also correspondingly stopped using $L_{depth}$ or $L_{albedo}$ . The experimental results show that reducing a certain embedding may lead to a better transfer of other identity features but have a large impact on the identity information represented by that embedding.

**Feature injection strategy:** For feature injection, we conducted experiments with three different strategies: (a) injecting the albedo embedding $e_{a}$ into the coarse feature mapper $M_{c}$ and the medium feature mapper $M_{m}$ and injecting the depth embedding $e_{d}$ into the fine feature mapper $M_{f}$ , (b) injecting the depth embedding $e_{d}$ into the coarse feature mapper $M_{c}$ and injecting the albedo embedding $e_{a}$ into the fine feature mapper $M_{f}$ and the medium feature mapper $M_{m}$ , and (c) injecting the ArcFace embedding $e_{arc}$ into the coarse feature mapper $M_{c}$ and the medium feature mapper $M_{m}$ and no further into the fine feature mapper $M_{f}$ . Experiments on strategies (a) and (b) show that our feature injection approach matches its semantic level. Experiments on strategy (c) show that there are a number of low-level semantic features in the ArcFace embedding $e_{arc}$ , and it is necessary to inject them into all three mappers.

**Training dataset size:** To investigate the influence of training set size on the model, we reduced the size of the training set from 27 k to 18 k and 9 k. Testing was still conducted on the initially selected testing set after training. The experimental results reveal that the effectiveness of our model is reduced with a decrease in the number of training set images. When the training set size is insufficient, the model exhibits signs of overfitting, particularly with a noticeable decline in the transfer performance of texture information.

### 4.6. Controllable Face Swapping

Distinguished from conventional face swapping methods, our model stands out by its exceptional capability to perform controllable face swapping, based upon the sufficient disentanglement of structure and texture. This is a pioneering breakthrough in the field of face swapping, as it empowers users with the freedom to choose their preferred identity transfer mode. When utilizing ControlFace for a face swapping project, users have the flexibility to decide whether to transfer the structure or texture of source faces to the target faces.

To enable a single face swapping model to seamlessly handle all of the four identity feature transfer modes, including complete identity transfer, structure-only transfer, texture-only transfer, and self-swapping, we designed a probabilistic framework that governs the transfer of structural and textural information during each training step, with both probabilities set at 0.7. Consequently, the probability distribution for each of the four transfer modes during a training step is 0.49, 0.21, 0.21, and 0.09, respectively.

**Figure 4:**
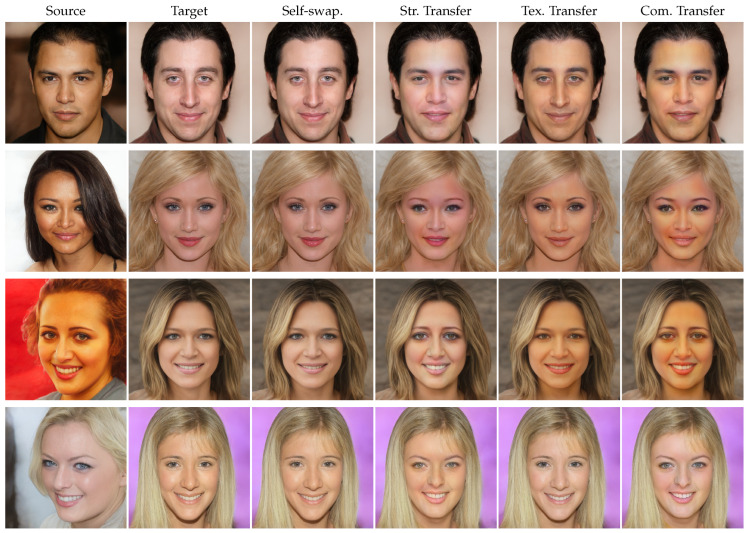
Qualitative results of controllable face swapping using our method.

**Qualitative results:** We show the generation results of each identity transfer mode in Figure 4, from which we can clearly make out the significant differences between the different modes.

When structure-only transfer is performed, the skin color, lip color, beard, and other texture information of the swapped face remain consistent with the target image, while the structure has a high similarity to that of the source face. In the field of virtual character creation, this mode plays a pivotal role in crafting lifelike virtual personas. It facilitates the fusion of unique face structures with pre-existing character templates while retaining the technologically synthesized skin and makeup. This is instrumental in generating diverse characters for video games, augmented reality experiences, and virtual worlds, enhancing the immersive quality of these digital environments.

While texture-only transfer is performed, the face structure of the swapped face is basically the same as the target face, but the texture information changes considerably, which is more consistent with the source face. In the field of beauty-themed applications, such as Photoshop and beauty filters in mobile applications, texture-only transfer can be utilized to enhance and refine individuals’ appearances in photos. Users can modify their skin texture and complexion to achieve a more desired and aesthetically pleasing look while retaining their original facial structure. This serves to meet the ever-evolving standards of beauty in the digital age, providing individuals with a means to perfect their selfies and photographs before sharing them on social media or elsewhere.

Our future research will concentrate on the further disentanglement of face structure and expression, as well as the separation of texture and lighting information. These plans have the potential to elevate the precision and interpretability of face swapping and editing techniques, which is able to give users a wider range of choices and higher-quality results.

### 4.7. Limitations

Despite the effectiveness of ControlFace, our method still has some limitations. Firstly, since our method uses the StyleGAN model as a baseline, dealing with low-quality facial images makes it challenging to obtain accurate latent codes in the W+ space, consequently hindering the generation of high-quality face swapping results. Secondly, our method requires training on a larger dataset to avoid overfitting issues. In future work, we aim to optimize our approach by addressing these aspects.

### 4.8. Broader Impact

Any realistic and high-quality face swapping technology, while providing services and experiences to users, may also give rise to certain societal and ethical implications, and our work is undoubtedly not an exception. This technology has the potential to maliciously exploit the facial features of ordinary individuals, leading to the leakage of personal privacy and infringement upon the citizen’s portrait rights. Additionally, the technology could be utilized for fraudulent activities, false advertising, political manipulation, and other malicious purposes, thereby having adverse effects on society.

These potential negative impacts of these face swapping methods underscore the necessity for facial forgery detection and facial privacy protection technologies. There is also an urgent need to establish rules and regulations governing the use of face swapping technology to ensure its corrective and responsible application in society. Rather than focusing solely on the potential malicious uses of face swapping technology or imposing a ban on research related to face swapping, our attention should be paid to applying face swapping technology in legitimate and compliant domains. We should be committed to enabling users to enjoy the benefits of this technology while helping them remain mindful of its potential risks.

## 5. Conclusions

In this paper, we propose ControlFace, a novel framework for face swapping. This method accurately disentangles the structure and texture of a source face and extracts them in the form of identity embeddings. We inject them into the feature mapper according to their semantic level and fully fuse them with the representation *w* of the target face in the W+ space of StyleGAN to generate high-fidelity, high-quality swapped faces. We realize controllable face swapping by extracting some of the identity embeddings from the source face and others from the target face. Extensive experiments and qualitative and quantitative comparisons with current mainstream methods demonstrate the superiority of our method in identity information transfer, attribute information protection, and controllable face swapping. 

## Figures and Tables

**Table 1:** **Quantitative results of identity transfer.** We compared our model with five competing methods in ArcFace Similarity and depth & albedo error for the ability of identity transfer. The best results are shown in bold. ↑ means higher is better, and ↓ means lower is better.

Method	Arc. Simi. ↑	Depth ↓	Albedo ↓
FaceShifter [1]	49.33	31.68	49.59
SimSwap [2]	52.03	32.32	48.49
HifiFace [3]	48.24	32.35	50.22
InfoSwap [43]	52.58	31.04	51.58
FaceDancer [44]	38.62	33.68	52.49
MegaFS [22]	48.49	33.44	48.18
HiRes [23]	48.81	30.71	42.58
**Ours**	**55.20**	**27.89**	**35.17**

**Table 2:** **Quantitative results of each face feature.** We measured the error of shape, texture, expression, pose, and lighting. ↑ means higher is better, and ↓ means lower is better.

Method	Shape ↓	Tex. ↓	Exp. ↓	Pose ↓	Light. ↓
FaceShifter [1]	2.07	5.34	**0.74**	**0.57**	1.08
SimSwap [2]	2.01	5.09	1.15	0.75	1.71
HifiFace [3]	1.75	4.95	1.23	0.63	2.14
InfoSwap [43]	2.01	4.91	1.38	2.41	1.93
FaceDancer [44]	3.45	7.19	0.77	0.75	**0.83**
MegaFS [22]	2.34	5.25	1.25	2.77	3.04
HiRes [23]	2.12	5.25	1.09	1.51	1.84
**Ours**	**1.26**	**3.12**	1.02	0.60	1.72

**Table 3:** **Quantitative results of StyleGAN-based face swapping methods for FaceForensics++ dataset.** We compared our model with two StyleGAN-based methods on the FaceForensics++ dataset to test the robustness of our method. ↑ means higher is better, and ↓ means lower is better.

Method	ID. Ret. ↑	Depth ↓	Albedo ↓
MegaFS [22]	90.31	34.03	47.91
HiRes [23]	88.23	33.14	44.05
**Ours**	**94.11**	**31.07**	**39.93**

**Table 4:** **Quantitative ablation study.** The comparison of different strategies of identity embedding selection, feature injection, and size of the training set. ↑ means higher is better, and ↓ means lower is better.

Method	Arc. Simi. ↑	Depth ↓	Albedo ↓
**Ours**	55.20	27.89	35.17
w/o $e_{arc}$	49.44	**27.80**	**34.58**
w/o $e_{d}$	55.97	28.62	35.07
w/o $e_{a}$	56.82	27.86	38.82
w/o $e_{d}$ & $e_{a}$	**57.06**	28.73	39.34
(a)	53.26	28.43	36.77
(b)	51.98	28.71	35.48
(c)	51.86	28.46	36.41
18 k-data	53.52	29.09	36.21
9 k-data	51.21	29.91	38.32

## Data Availability

Our code will be publicly available at https://github.com/ZhangXH227/ControlFace (accessed on 12 February 2023). Publicly available datasets were analyzed in this study. This data can be found here: CelebAMask-HQ: https://github.com/switchablenorms/CelebAMask-HQ (accessed on 15 May 2019).
